# Health insurance fraud detection by using an attributed heterogeneous information network with a hierarchical attention mechanism

**DOI:** 10.1186/s12911-023-02152-0

**Published:** 2023-04-06

**Authors:** Jiangtao Lu, Kaibiao Lin, Ruicong Chen, Min Lin, Xin Chen, Ping Lu

**Affiliations:** 1 School of Computer and Information Engineering, Xiamen University of Technology, Xiamen, 361024 China; 2 School of Electromechanical and Information Engineering, Putian University, Putian, 351100 China; 3 School of Economic and Management, Xiamen University of Technology, Xiamen, 361024 China

**Keywords:** Health insurance, Fraud detection, Heterogeneous graph, Graph neural network

## Abstract

**Background:**

With the rapid growth of healthcare services, health insurance fraud detection has become an important measure to ensure efficient use of public funds. Traditional fraud detection methods have tended to focus on the attributes of a single visit and have ignored the behavioural relationships of multiple visits by patients.

**Methods:**

We propose a health insurance fraud detection model based on a multilevel attention mechanism that we call MHAMFD. Specifically, we use an attributed heterogeneous information network (AHIN) to model different types of objects and their rich attributes and interactions in a healthcare scenario. MHAMFD selects appropriate neighbour nodes based on the behavioural relationships at different levels of a patient’s visit. We also designed a hierarchical attention mechanism to aggregate complex semantic information from the interweaving of different levels of behavioural relationships of patients. This increases the feature representation of objects and makes the model interpretable by identifying the main factors of fraud.

**Results:**

Experimental results using real datasets showed that MHAMFD detected health insurance fraud with better accuracy than existing methods.

**Conclusions:**

Experiment suggests that the behavioral relationships between patients’ multiple visits can also be of great help to detect health care fraud. Subsequent research fraud detection methods can also take into account the different behavioral relationships between patients.

## Background

**Fig. 1:**
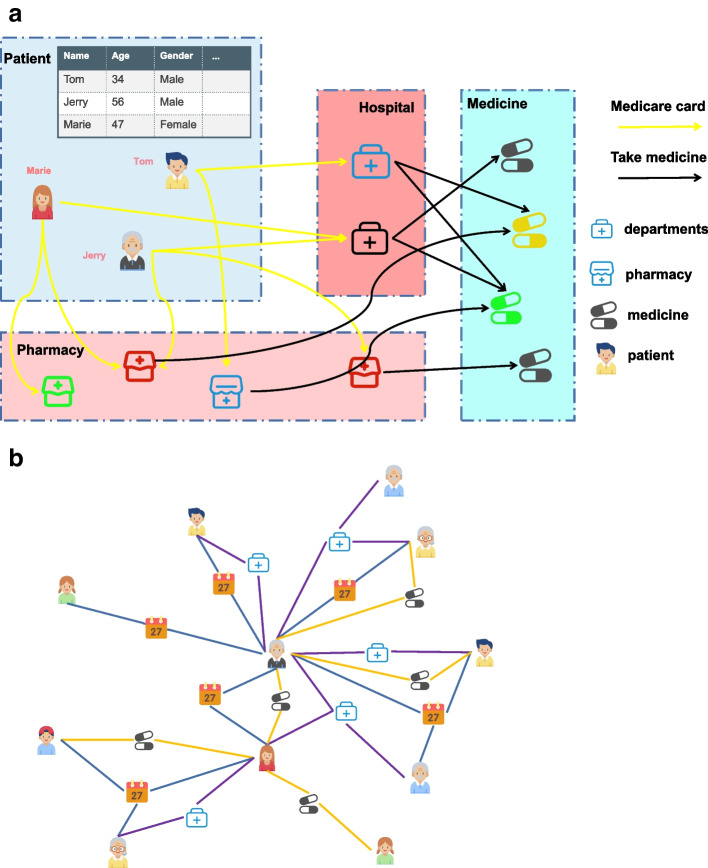
AHIN of the scenario of medical payment service: **a** Scenario of medical payment service; **b** Heterogeneous Network

The popularisation of health insurance has provided many people with easy access to healthcare and medical protection for the public. In various countries, the rapid development of the medical services sector is inseparable from governmental support for healthcare. In the United States, healthcare generated about 3.5 trillion USD in 2017 [1], of which Medicare contributed about 20% or 702 billion USD [2]. However, health insurance fraud is also increasing, which poses a serious threat to the proper use of public funds. According to a survey by the Global Healthcare Anti-Fraud Network, approximately 260 billion USD is lost globally to health insurance fraud each year, which is equivalent to 6% of global healthcare spending [3]. According to the Coalition Against Insurance Fraud, the annual financial losses due to Medicare fraud in the United States is equivalent to 3–10% of healthcare expenditures [3], or 21-70 billion USD. A large number of medical claims are made every day and fraud can be committed in many ways, such as the illegal administration of drugs and consumables, purchase and resale of drugs and falsification of bills for medical services. Such fraud seriously harms the interests of users and service providers. In response, researchers have been developing methods to detect health insurance fraud. In China, 339 million USD was recovered from cases of health insurance fraud in 2020 alone [4]. In 2020, 1 USD = 6.58 RMB [5]. The main approach has been manual detection by government-organised experts, which is effective but requires a major expenditure of time and effort. It relies on sufficient a priori knowledge and it cannot detect incidents of health insurance fraud consistently and automatically. In addition, there are many types of health insurance fraud and methods relying on manual detection can have difficulty coping with complex and changing patterns. To address these limitations, another approach is to apply machine learning to finding incidents of health insurance fraud automatically. This approach finds outliers in the data, which represent incidents of fraud and uses data mining to identify special patterns of anomalous data that differ significantly from the rest of the data. Most methods based on machine learning extract statistical features from the user data such as the user’s access trajectory, behaviour and cost. These methods then use such statistical features for prediction and classification by neural networks, random forests or other classifiers. Such methods rarely make full use of the interaction between users. However, many entities (i.e. objects) are involved in healthcare, such as patients, hospitals, departments and drugs. There are rich interactions between these objects that can be very important for detecting health insurance fraud. Some researchers have started to use graph embedding to incorporate user interactions. However, most graph embedding based models are black-box models and solving a specialised domain problem such as health insurance fraud detection often requires a certain degree of interpretability. In addition, previous approaches to health insurance fraud detection have tended to focus only on the characteristic attributes of a single visit. The behavioural attributes of multiple visits have been neglected. Such behavioural relationships can be modelled by a heterogeneous graph approach, which can also be used to find anomalies in the topology of the graph neural network. Graphs are widely used to model complex relationships between objects in many fields [6], including computer vision [7], natural language processing, anomaly detection [8], academic network analysis and recommendation systems [9]. Figure 1(a) shows a general scenario of medical treatment, which has several important objects such as the patient, doctor, hospital, department and medicine. In addition to attribute information, such objects also possess rich interactive information, such as the patient going to the hospital on a certain date or drugs being prescribed to a patient by a particular department. Integrating the patient’s attribute information requires expanding the traditional heterogeneous information network (HIN) into an attributed HIN (AHIN) so that the objects in the heterogeneous information network can possess characteristics. Figure 1(b) shows the architecture of the AHIN for the medical treatment scenario, which contains the objects and their interactions. However, there are still several challenges. First, if health insurance data are modelled by an AHIN, the complexity of the data will lead to a large number of useless nodes in the graph. A graph neural network works by representing a learning task as the propagation and aggregation of attributes among nodes. A large number of noisy nodes will inevitably affect the results. Second, fraudulent users need to be distinguished from innocent patients. The results of the detected need to be interpretable considering its potential application in a professional setting. Finally, distinguishing which behavioural relationship of a medical visit is important for analysis is another challenge. We propose a health insurance fraud detection model based on a multilevel attention mechanism that we call MHAMFD. The basic idea of MHAMFD is to enhance the user representation by considering interactions between objects through neighbour nodes obtained from multilevel behavioural relationships in an AHIN. Specifically, we build bridges between patient nodes through behavioural relationships for medical visit scenarios. The different behavioural relationships are used to construct a medical multi-relational graph network. Meanwhile, we propose a multi-level graph neural network which is used to model multiple behavioural relationships within the network for fraud detection. The model has several advantages: (1) Using different levels of behavioural relationships in a way to select appropriate neighbour nodes to form different isomorphic graphs. This can effectively reduce the graph structure and can integrate the semantic information interwoven with multiple behavioural relationships [10]. It is also possible to obtain a comprehensive fraud detection result. (2)Due to the effectiveness of attention mechanism in various machine learning tasks [11, 12], we design a hierarchical attention mechanism. The first level of intra-relationship aggregation (intra-relation AGG) is an attention mechanism that can effectively associate different attributes of different neighbours. The second level of inter-relationship aggregation (inter-relation AGG) can effectively correlate different behavioural relationships of patients. The third layer of hierarchical behavioural aggregation (hierar-relation AGG) can aggregate complex semantic information from the interweaving of different levels of behavioural relationships of patients. This effectively reflects which levels of behavioural relationships are more important for the final task. This attention-based model can improve interpretable results to provide more insights about the health care fraud detection task and outcomes. In summary, the contributions of our study can be summarised as follows:

- To the best of our knowledge, MHAMFD is the first to sample multiple behavioural relationships among neighbours to obtain a heterogeneous network of healthcare attributes. The interactions between different types of objects are learned by capturing structural information between objects in real healthcare scenarios.
- We designed a hierarchical attention mechanism for automatically learning the importance of different patient access behaviours in the healthcare domain for fraud detection.
- Extensive experiments with two real datasets showed that MHAMFD performed better at fraud detection than existing graph representation learning methods.

### Related work

#### Health insurance fraud detection

Health insurance fraud has a significant impact on healthcare and daily life. Early research on fraud detection focused on a rule-based approach. Bauder and Khoshgoftaar [13] concluded that health insurance fraud has some obvious behavioural signals, which can be detected by defining some combination of rules. Because of the simplicity and interpretability of rule-based approaches, they have long been used for fraud detection [14]. However, designing such rules depends on a priori knowledge. In addition, there are so many ways to commit health insurance fraud that this approach has difficulty in dealing with complex and changing patterns. Recent methods have started using machine learning to find the intrinsic patterns in data indicating fraud automatically. Current detection methods can be classified into two general categories [15, 16]: unsupervised and supervised learning. Methods based on unsupervised learning depend on the data distribution. Machine learning is used to find outliers in the data, which indicate fraud and data mining is used to find abnormal data that differ significantly from general data for a specific mode. However, such methods are limited because they examine fraudulent behaviour according to a specific model. For example, Zhang and He [17] examined outliers in the treatment of diseases and the costs incurred to identify fraud related to falsified expenses. However, health insurance-related businesses are becoming increasingly detailed and fraudulent behaviour is becoming increasingly complex, changeable and concealed. New patterns of fraud continue to appear and anomaly detection algorithms based on fixed patterns lack immunity to these new patterns. Thus, fraud detection methods based on fixed patterns have difficulty with meeting current needs [18]. Predictive models based on supervised learning require large amounts of labelled data, but the data of actual scenarios are generally unlabelled and less data are available to protect patient privacy. Bauder and Khoshgoftaar [19] used random forest to classify unbalanced data. Pandey et al. [20] proposed using rule-based scoring systems, logistic regression models and decision trees, all of which strongly rely on a large amount of training data.

#### Graph-based learning

Our proposed model is related to graph-based methods. Network embedding is an effective method for modelling graph structures. Representation learning is performed by mapping the network nodes into a low-dimensional vector space. Then, low-dimensional dense vectors are used to represent any node in the network, which can be flexibly applied to different data mining tasks. Many early studies focused on representation learning with homogeneous networks, where existing deep models were combined with network features to learn feature representations of nodes or edges. DeepWalk [21] combines random walk and the skip-gram model to learn network node representation. LINE [22] adds a second-order similarity based on first-order neighbour similarity to learn strongly distinguished node representation for large scale sparse networks. SDNE [23] uses a deep autoencoder to extract nonlinear characteristics of a network structure. However, homogeneous network modelling often extracts only part of the information of an actual interactive system or does not distinguish the heterogeneity of objects and their relationships, which results in irreversible information loss [24, 25]. In contrast, heterogeneous network modelling provides two benefits. First, a heterogeneous network is an effective tool for fusing information: not only different types of objects and their interactions but also information from heterogeneous data sources [26]. Second, the coexistence of multiple types of objects and relationships in heterogeneous networks contains rich structural and semantic information, which provides a precise and interpretable new way of discovering hidden patterns. Further methods have been proposed that use meta-paths [27] to explore the network topology and node characteristics. Some models guide the selection of neighbours by defining multiple meta-paths in a heterogeneous graph. However, such models only consider the importance of nodes and meta-paths and ignore the structural information of different levels of relationships intertwined in heterogeneous graphs. This can lead to underutilisation of structural information in the graph. In our study, one of our aims was to use the structural information obtained from the hierarchy of different behavioural relationships to learn the implicit interactions among objects in the healthcare services domain to improve the fraud detection accuracy.

### Preliminaries

A HIN is an information network that contains multiple types of objects and multiple types of links [6]. Because actual patient data have information on certain attributes, the HIN can be extended into an AHIN to integrate these attributes. Here we define several terms necessary for understanding the explanation of our proposed model.

#### Heterogeneous graph

A heterogeneous graph is a special information network that can be denoted as a graph $G=\{V, \varepsilon , X\}$ comprising the set of nodes *V*, set of links and attribute information matrix $\textrm{X} \in R^{|v| \times k}$. A heterogeneous graph is associated with a node type mapping function $\emptyset : V \rightarrow A$ and link type mapping function $\varphi : \varepsilon \rightarrow R$, where *A* and *R* denote the sets of predefined object types and link types, respectively. Normally, a network can be considered a heterogeneous graph when the number of node types |*A*| and link types |*R*| meet the condition $|A|+|R|>2$. Otherwise, it is a homogeneous graph.

#### Multiple behavioural relationship paths

The behavioural relationship paths in the medical domain can be used to capture rich semantic information for the heterogeneous graph. A behavioural relationship path is an abstract sequence of node types connected by link types that is denoted in the form of $A_{1} {\mathop {\longrightarrow }\limits ^{M_{1} R_{1}}} A_{2} {\mathop {\longrightarrow }\limits ^{M_{2} R_{2}}} A_{3} {\mathop {\longrightarrow }\limits ^{M_{3} R_{3}}} \cdots {\mathop {\longrightarrow }\limits ^{M_{l} R_{l}}} A_{l+1}$, which can be abbreviated as $A_{1} A_{2} A_{3} \cdots A_{l+1}$. It describes a composite relationship $M R=M_{1} R_{1}{ }^{\circ } M_{2} R_{2}{ }^{\circ } \cdots ^{\circ } M_{l} R_{l}$ between node types $A_{1}$ and $A_{l}$, where $^{\circ }$ denotes the composition operator of relationships. Depending on the number of edges connecting two adjacent patient nodes, the behavioural relationship can be classified as single-level or multilevel.

#### Neighbours according to multiple behavioural relationships

For a given user *u* in a HIN with a given attribute, the neighbours of the behavioural relationship paths are defined as the aggregate neighbour set for a given behavioural relationship path of the user *u* in the AHIN.

#### Multi-relational medical graph

A heterogeneous medical graph is a HIN extracted from health insurance data. To prevent the loss of heterogeneous information between different types of nodes and to reduce the size of the graph, we can map this to multi-relationship medical graph that only keeps the patient nodes. This converts the heterogeneous graph to an isomorphic graph while preserving the rich interaction information.

#### Node selection

**Fig. 2:**
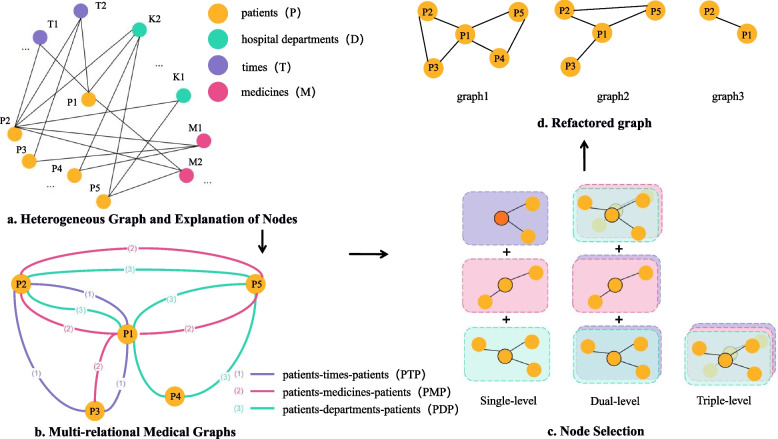
Process of decomposing and reconstructing heterogeneous graphs

The different levels of impurities in different behavioural relationships in a multi-relationship graph will affect the embedding results. There may be cases where the neighbours of a patient node are connected by different numbers of edges. To analyse which behavioural relationship or composite behavioural relationship has a greater impact on identifying health insurance fraud, we can first sample different behavioural relationships and then find neighbour nodes connected with the same behavioural relationship. Figure 2 shows the process of decomposing and reconstructing a heterogeneous graph. As an example, Phase b in Fig. 2 shows that the p2, p4 and p5 nodes are connected to the patient node p1 through the single-level behavioural relationship PDP. Only the p2 node is connected by the multilevel behavioural relationship PDTMP.

#### Health insurance fraud detection problem with an AHIN

The health insurance fraud detection problem can be defined by modelling different objects and their interactions in a real medical treatment scenario as the AHIN $G=\{V, \varepsilon , X\}$. In our experiments, the patient node set was a subset of the node set denoted as $U \subset V$. In the dataset, each patient $u \subset V$ had the label $Y u \in \{0,1\}$ to indicate whether the patient was fraudulent. For a given AHIN $G=\{V, \varepsilon , X\}$ and training set $D=\{(u, Y u)\}$, the ultimate goal is to predict the probability that a patient in the test set is fraudulent.

## Methods

### Theory

**Fig. 3:**
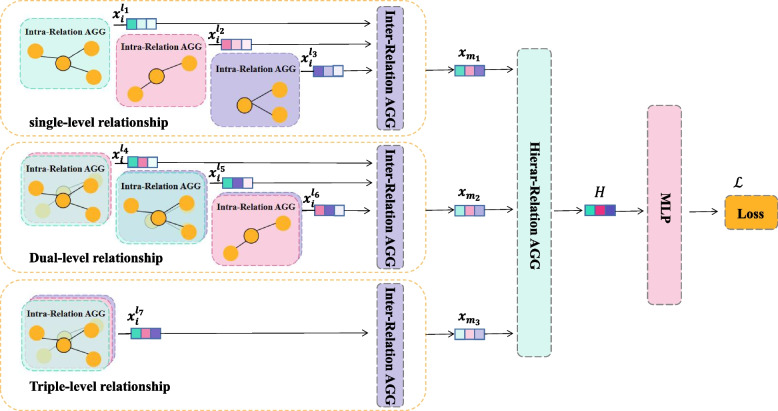
Architecture of the MHAMFD model

Here, we present the proposed model MHAMFD, which uses a hierarchical attention mechanism for fraudulent user detection. Figure 3 shows the general architecture of MHAMFD. The basic concept is to enhance the user’s representation by making full use of interactions with neighbour nodes based on multilevel behavioural relationships in an AHIN.

#### Observations of real data

**Fig. 4:**
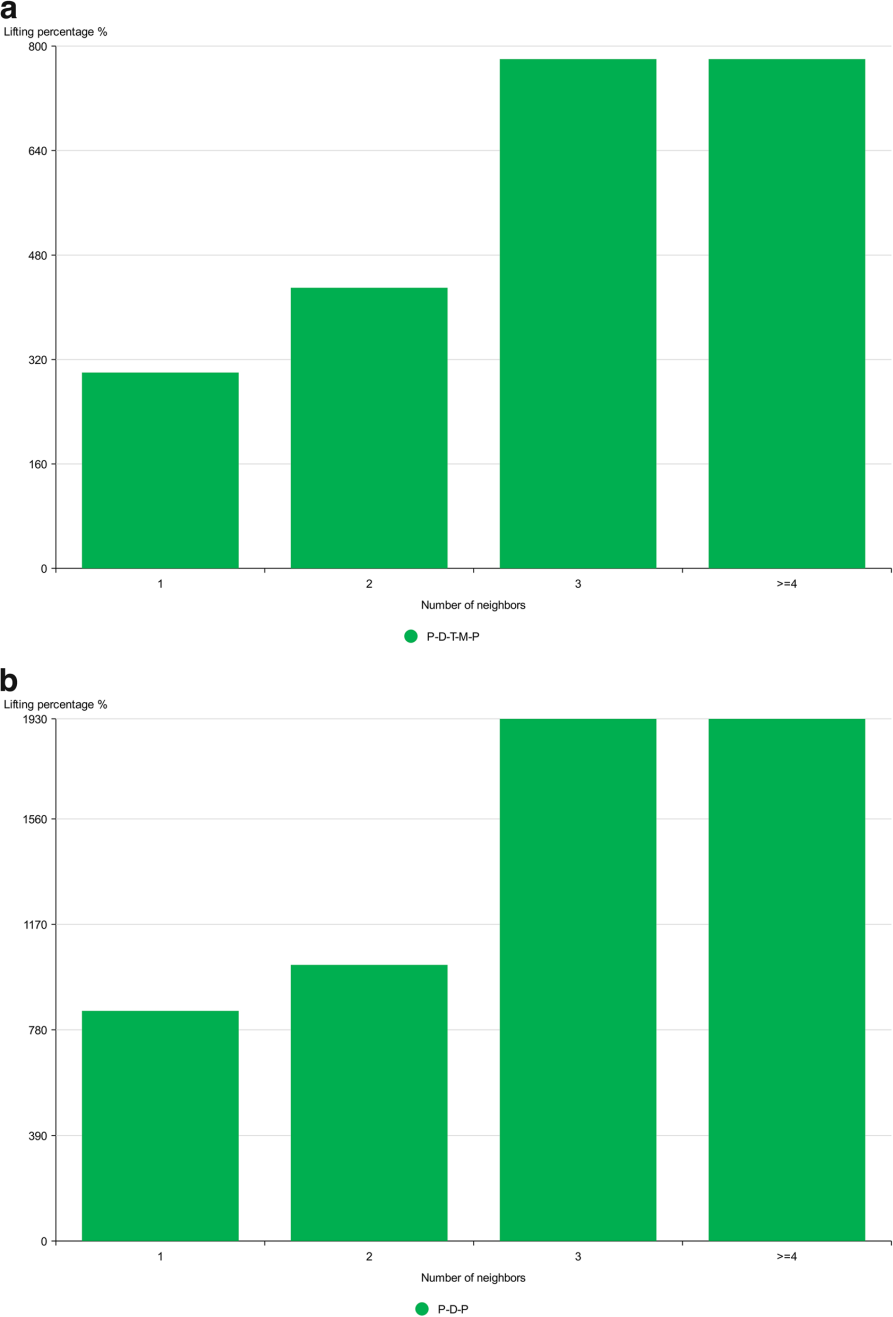
Lifting percentages of fraud rate in users with different amount of Medicare fraudster neighbours against users without any Medicare fraudster neighbour in two behaviour relationships: **a** Behavioural relationship PDTMP; **b** Behavioural relationship PDP

Intuitively, fraudulent users of health insurance tend to cluster closely through different types of interactions. For example, in the AHIN shown in Fig. 1(a), fraudulent users tend to make medical visits to specific departments or use health insurance cards on a large scale at a uniform point in time. To aggregate the diverse behavioural relationships of fraudulent users, we conducted experiments on a real dataset. First, we collected path-based neighbours for each user according to two different paths: PDP (i.e. the user visited the same department) and PDTMP (i.e. the user visited the same department on the same day, and both users picked up the same medication). For each path, we counted the number of neighbours who are users and divided all users into groups based on the number of fraudulent neighbours. The probability of health insurance fraud was calculated for each group. Figure 4 shows the percentage boost in fraud rate for the user groups with and without fraudulent neighbours for both paths. The different paths produced different percentage boosts. This confirmed to us that different behavioural relationships have different levels of importance to users, which can be captured by an attention mechanism.

#### Intra-relational aggregation

To better guide the embedding of a multi-relational graph neural network, we propose aggregation based on different levels of behavioural relationships. The overall aggregation process of MHAMFD is divided into three parts depending on the type of relationship: intra, inter and hierarchical. For the intra-relational aggregation process, the selected neighbours of a node play different roles in node embedding based on the behavioural relationship. In other words, each neighbour node has a different importance. For example, in the neighbour set based on the behavioural relationship PDP, the feature similarity varies between the target node and each neighbour node. This affects the feature representation of the target node that can be learned from the neighbour nodes. Therefore, we use the self-attention mechanism in the intra-relational aggregation process. We aggregate the embedding representations of meaningful neighbours, which forms node embedding representations that are specific to a certain behavioural relationship. Concretely, we use the self-attention mechanism to learn the importance of each neighbouring node to the target node. Corresponding weights are assigned to different neighbour nodes. Given a pair of nodes (*i*, *j*) connected by the behavioural relationship *l*, we can define the importance of the neighbour node *j* to the target node *i* as $E_{i j}^{l}$. The importance of the node pair (*i*, *j*) based on the relationship *l* can be expressed as follows,

1 $$\begin{aligned} E_{i j}^{l}=A t t_{i n t r a}\left[ x_{i}, x_{j}; l\right] \end{aligned}$$

where $x_{i}$ and $x_{j}$ respectively represent the embeddings of nodes *i* and *j*, and *l* represents the behavioural relationship connecting the two nodes. $A t t_{i n t r a}$ represents the deep neural network that implements the attention mechanism for intra-relational aggregation. For a given behaviour relationship *l*, all node pairs share $A t t_{i n t r a}$. This is because the neighbours of all target nodes are selected by the same behavioural relationship. However, because of the asymmetry of the heterogeneous graph, $E_{i j}^{l}$ is asymmetric. This means that the importance of node *i* to node *j* differs from the importance of node *j* to node *i*. The importance of a node depends on its characteristics. When the target node changes, $E_{i j}^{l}$ also changes because the neighbour nodes of the target node based on the behavioural relationship *l* have changed. This can be expressed as $j \in N_{i}^{l}$ and $i \in N_{j}^{l}$, where $N_{i}^{l}$ represents the neighbour of node *i* based on the behavioural relationship *l*. Afterwards, normalisation is performed with the *Softmax* function. This facilitates the embedding of aggregated neighbours, which is expressed by,

2 $$\begin{aligned} \alpha _{i j}^{l}={softmax}_{j}\left( E_{i j}^{l}\right) =\frac{\exp \left( {LeakyReLU}\left( E_{i j}^{l}\right) \right) }{\sum _{p \in N_{i}^{l}} \exp \left( {LeakyReLU}\left( E_{i p}^{l}\right) \right) } \end{aligned}$$

The attention mechanism in intra-relation aggregation is a single-layer feedforward neural network. Here, we need to add LeakyReLU function for nonlinear activation and we set the slope to 0.2. Calculating the importance of neighboring nodes using LeakyReLU, we can pay more attention to neighboring nodes that are more positively related to the target node. The weight coefficients of (*i*, *j*) depend on their characteristics. The weight coefficient $\alpha _{i j}^{l}$ is also asymmetric, which means that the contributions of nodes *i* and *j* to each other also differ. This is because the target node changes, which changes the neighbour domain. Nodes *i* and *j* have different neighbours, so the normalisation term (denominator) differs. After the importance of all neighbouring nodes for the target node *i* is obtained, we can aggregate the attribute features of all neighbours connected by the behavioural relationship *l* with the corresponding coefficients,

3 $$\begin{aligned} x_{i}^{l}=\sigma \left( \sum \nolimits _{j \in N_{i}^{l}} \alpha _{i j}^{l} \cdot x_{j}\right) \end{aligned}$$

where $x_{i}^{l}$ is the embedding representation learned by node *i* for the behavioural relationship *l* and $\sigma$ is the activation function. Because heterogeneous graphs have scale-free characteristics, the variance of the graph data is relatively large. To reduce the variance, we can use multiple attention heads to make the training process more stable. Specifically, we repeat the attention mechanism *k* times and we concatenate the embeddings learned each time,

4 $$\begin{aligned} x_{i}^{l}=\mathop \Vert _{k=1}^{K} \sigma \left( \sum \nolimits _{j \in N_{i}^{l}} \alpha _{i j}^{lk} \cdot x_{j}^{k}\right) \end{aligned}$$

Given the feature vector *x* and the set of behavioral relationships $\left\{ l_1, l_2, \ldots , l_m\right\}$, the Intra-relational attention of MHAMFD generates *m* behavior-specific vector representations of the target node, which are denoted as $\left\{ x_{l_1}, x_{l_2}, \ldots , x_{l_m}\right\}$.

#### Inter-relational aggregation

Node embeddings specific to a certain behavioural relationship can only reflect node information one way. For a more comprehensive representation of node embeddings, information about these behavioural relationships needs to be fused. For health insurance fraud detection, each behavioural relationship has a different impact on node embedding. The behavioural relationships connecting target nodes in the heterogeneous graph have different meanings. For example, the set of patient nodes selected by the behavioural relationship PDP differs from the set of patient nodes selected by PTP and the embeddings learned by both are not the same. These have different degrees of importance for determining whether the target patient is fraudulent. Therefore, we use an attention mechanism in the inter-relational aggregation process to automatically learn the weights of different behavioural relationships and aggregate them. This is done by nonlinear transformation of the node vectors that have undergone intra-relational aggregation and then averaging them. The importance of each behavioural relationship is given by,

5 $${\omega_{l_i}}=\frac1{\vert V\vert}\sum\nolimits_{i\in V}q_0^T\cdot\tanh\left(W_0\cdot x_i^l+b_0\right)$$

where ${\omega_{l_i}}$ is the importance of a behavioural relationship, $q_{0}$ is the attention vector, $W_{0}$ is the weight matrix and $b_{0}$ is the bias vector. For a meaningful comparison of the importance of different behavioural relationships, all of the above parameters are shared. Then, normalisation is performed by the *Softmax* function. The weight coefficient can be obtained as follows,

6 $$\begin{aligned} {\beta _{l_i}}={\text {softmax}}\left( {\omega _{l_i}}\right) =\frac{\exp \left( {\omega _{l_i}}\right) }{\sum \nolimits _{i=1}^{l} \exp \left( {\omega _{l_i}}\right) } \end{aligned}$$

After the attention weights are obtained for each behavioural relationship, the attention weights of each behavioural relationship and the embedding representations between relationships are weighted and summed,

7 $$x_m=\sum_{i=1}^l{\beta_{l_i}}\cdot {x_{l_i}}$$

In summary, for a given embedding of nodes specific to a certain behavioural relationship, the contribution of each behavioural relationship can be expressed by Eq. (7). $A t t_{i n t e r}$ represents the deep neural network that performs the attention mechanism. In summary, given a group behavior-specific vector embedding as input, the contribution of each behavioral relationship can be denoted as follows,

8 $$\begin{aligned} \left( \beta _{l_1}, \beta _{l_2}, \cdots , \beta _{l_m}\right) =A t t_{i n t e r}\left( x_{l_1}, x_{l_2}, \cdots , x_{l_m}\right) \end{aligned}$$

After getting the importance of behavioral relationships, the behavioral relationships of each level are aggregated and generated *n* level-specific vector embedding, denoted as $\left\{ x_{m_1}, x_{m_2}, \ldots , x_{m_n}\right\}$.

#### Hierarchical relational aggregation

We perform a clipped reconstruction of the nodes and edges in the multi-relationship graph for health insurance fraud detection. Finally, isomorphic graphs are obtained with only single rows representing single-level, two-level and three-level behavioural relationships. After the intra-relational and inter-relational aggregations, we have obtained graph embedding representations of the target nodes based on three levels of behavioural relationships. Our observations of a real health insurance dataset indicated that the different levels of behavioural relationships are relevant for fraud detection. For more comprehensive node embedding representation, the information of the different levels of behavioural relationships needed to be fused. Therefore, we designed a relational aggregation mechanism that captures the semantic information from the hierarchy of the behavioural relationships. For a given set of embeddings, if the degree of influence of different levels of behavioural relationships on the final task is added, then this can be represented as follows,

9 $$\begin{aligned} \left( \gamma _{m_1}, \gamma _{m_2}, \cdots , \gamma _{m_n}\right) =A t t_{\text{ hierar } }\left( x_{m_{1}}, x_{m_{2}}, \cdots , x_{m_{n}}\right) \end{aligned}$$

10 $$\begin{aligned} \lambda _{m_i}=\frac{1}{|N|} \sum \nolimits _{i \in N} q_{1}^{T} \cdot {tan} h\left( W_{1} \cdot x_{m_i}+b_{1}\right) \end{aligned}$$

11 $$\begin{aligned} \gamma _{m_i}={softmax}\left( \lambda _{m_i}\right) =\frac{\exp \left( \lambda _{m_i}\right) }{\sum \nolimits _{i=1}^{n} \exp \left( \lambda _{m_i}\right) } \end{aligned}$$

where $\lambda _{m_i}$ is the level of importance of a behaviour, $q_{1}$ is the attention vector, $W_{1}$ is the weight matrix and $b_{1}$ is the bias vector. Specifically, a larger $\lambda _{m_i}$ means that the behaviour at level $m_i$ is more important for the final task. Finally, we need to aggregate the information contained at each level to obtain the final node embedding *H*,

12 $$\begin{aligned} H=\sum \limits _{i=1}^{n} \gamma _{m_i} \cdot x_{m_i} \end{aligned}$$

We can apply the final embedding *H* to different downstream tasks. We can use cross-entropy as the loss function and optimise the model weights by backpropagation of the minimisation function. The cross-entropy is expressed as,

13 $$\begin{aligned} \mathcal {L}=-\sum \limits _{s \in y_{S}} Y_{S} \ln \left( W \cdot H^{s}\right) \end{aligned}$$

where $y_{S}$ is the set of node indices with labels, $H^{s}$ and $Y_{S}$ are the embeddings of labelled nodes and the corresponding labels and *W* is the classifier. We can use the hierarchical attention mechanism to aggregate information at each level and obtain meaningful node embeddings.

### Experimental

We conducted experiments to evaluate the proposed model with real datasets, compare it against some baseline methods and use visualisation methods for a more intuitive presentation of the results. Here, we briefly describe the basic experimental setup. We performed ablation experiments to compare the effects of different modules. Finally, we analysed the effect of the hierarchical attention mechanism on the final task.

#### Datasets

We used two real datasets from a certain municipal health insurance bureau in China in 2018: Medical-1 and Medical-2. The fraud samples in the datasets were the same: patients detected because of abnormal kidney disease, repeated prescriptions, prescription of senile dementia drugs in their 80s and simultaneous outpatient hospitalisation. The main difference was that Medical-1 contained balanced samples with a ratio of positive to negative samples of 1:2. Medical-2 contained unbalanced samples with a ratio of positive to negative samples of about 1:70. Table 1 presents the detailed information on the two datasets.

**Table 1:** Dataset used in the experiment

Dataset	Node	Positive samples	Negative samples	Behavior-Relationship
Medical-1	Patient(P):440	152	288	PDP
	Time(T):351			PTP
	Medicine(M):2328			PMP
	Department(D):708			PDTP
				PDMP
				PTMP
				PDTMP
Medical-2	Patient(P):10647	152	10495	PDP
	Time(T):364			PTP
	Medicine(M):4718			PMP
	Department(D):2751			PDTP
				PDMP
				PTMP
				PDTMP

#### Baselines

We compared the performance of our proposed MHAMFD against multiple advanced methods, including those based on graph structures and graph neural networks. The following baseline models were considered:

- Metapath2vec [28]: A heterogeneous graph embedding method that combines random walk guided by a meta-path with a skip-gram model. We tested different meta-paths to obtain the best performance.
- GCN [29]: A variant of a graph neural network designed for isomorphic graphs. It is a semi-supervised graph convolutional network. We tested all paths to obtain the best performance.
- GAT [30]: A variant of a graph neural network designed for isomorphic graphs. It is a semi-supervised neural network with an attention mechanism. We tested all paths to obtain the best performance.
- HAN [31]: A heterogeneous graph neural network for graph embedding. This model learns node embeddings specific to a meta-path from different isomorphic graphs and uses a two-level attention mechanism to aggregate them into a vector embedding that represents each node in the network.
- MHAMFD : The proposed model. It is a semi-supervised graph neural network that uses a nested aggregation mechanism to simultaneously learn the importance of each object in a heterogeneous graph.
- MHAMFD$_{\text{ hierar } }$: A variant of MHAMFD that removes the hierarchical attention mechanism and assigns the same weight to each level of behavioural relationships.
- MHAMFD$_{\text{ single } }$: A variant of MHAMFD that only uses single-level behavioural relationships for node selection.
- MHAMFD$_{\text{ multi } }$: A variant of MHAMFD that use two- and three-level behavioural relationships for node selection.

#### Parameter settings

For Metapath2vec, we conducted a random walk for the patient nodes in the order of patient $\rightarrow$ medicine $\rightarrow$ patient $\rightarrow$ date $\rightarrow$ patient $\rightarrow$ hospital department $\rightarrow$ patient. Each patient node was randomly sampled 20 times and the number of negative samples was set to 5. For the semi-supervised graph neural networks (i.e. GCN, GAT and HAN), we used the same training set, validation set and test set for each to ensure fairness. For a fair comparison, we set the embedding dimension to 64 for all of the above algorithms. For MHAMFD, we used the Adam optimisation algorithm and set the learning rate to 0.005, number of attention heads to 8. We set the dimensions of the attention vectors and to 128. In order to cope with the small training set, regularization is widely used in the model to prevent over-fitting. In training, we adopt L2 regularization and set the regularization parameter $\lambda$ to 0.001. We introduce L2 regularization to make some restrictions. When the weight parameters are updated, the absolute values of the weight parameters are continuously reduced to prevent over-fitting. Meanwhile, we set the dropout parameter to 0.6, which makes the data in each batch inconsistent, so it can be simply regarded as many different models for training, so as to get more robust weights, achieve multi-model fusion, improve the generalization of models and reduce the over-fitting rate of models. Also, we set an early stop parameter patience to 50. If the loss of the model does not decrease and the accuracy does not improve in 50 consecutive training cycles, the training will be terminated early. In this way, the problems of too long training and over-fitting can be prevented.

## Results and Discussion

### Node classification

To evaluate the effectiveness of MHAMFD, we started with node classification. First, we conducted experiments with Medical-1 to compare the performances of different models. We divide the dataset into three subsets: training, validation and test. The F-score (f1) and accuracy (acc) were used as validation metrics. We used end-to-end training for all models. A multilayer perceptron was connected to the end of a model, which was then optimised by using the cross-entropy loss function. Table 2 presents the node classification results. We can see that the performance of our proposed MHAMFD model is better than the baseline. Our MHAMFD model has statistical significance in all three evaluation indexes (two-tailed t test, $\alpha$ = 0.01, *P* < 0.01). The overall effectiveness of Metapath2vec was quite different from that of the other models. This means that only using structural information for a small number of samples did not result in learning better embedding representations. GAT, GCN and HAN all used meta-paths to find groups of identical behavioural trajectories. In contrast, MHAMFD mined the same behavioural trajectories to obtain different levels of behavioural relationships. Differences between patient nodes were detected by mining the deep semantic information of heterogeneous graphs through multilevel behavioural relationships. On average, f1 was 6% higher for MHAMFD than for the other models. This indicates that single-level relational paths are not enough to explore the structure of heterogeneous graphs and that the structural information of different levels of relationships needs to be mined.

**Table 2:** Classification effectiveness with balanced sample nodes

Train:Val:Test	Metrics	Metapath2Vec	GAT	GCN	HAN	MHAMFD_hierar_	MHAMFD
1:1:3	f1	0.5595	0.7979	0.7914	0.791	0.8197	0.8694
	acc	0.7196	0.8523	0.8523	0.8598	0.8611	0.8961
2:1:2	f1	0.5964	0.7273	0.7547	0.7455	0.8354	0.8566
	acc	0.7386	0.8295	0.8523	0.8409	0.8678	0.8905
3:1:1	f1	0.6349	0.72	0.7772	0.7407	0.8123	0.8439
	acc	0.7386	0.8409	0.8371	0.8409	0.8423	0.8757

### Anomaly detection

Next, the above models were evaluated in terms of anomaly detection. We used Medical-2 for the experiment. We used the weighted cross-entropy loss function for training and obtained the embedding representation of each model with different training sets. Table 3 presents the anomaly detection results. Unlike for Medical-1, which had balanced samples, in this experiment the effectiveness of GCN was greatly reduced. This is because GCN treats all neighbours equally during aggregation, so the large proportion of non-fraudulent patients introduced a large amount of noise. The representations learned by GCN did not cope well with the task of anomaly detection. Overall, Metapath2vec was more effective with a large number of samples with rich structural information than with a small number of samples. Metapath2vec includes some structural information of the network but ignores the characteristic information of nodes, so the performance was generally mediocre. Both MHAMFD and HAN have an attention mechanism that allows nodes to distinguish the importance of neighbours. However, HAN cannot extract different levels of complex behavioural relationships, at least within the medical domain. These different levels of behavioural relationships have a significant impact on node embedding representation. MHAMFD considers more complex semantic information that it obtains from different levels of behavioural relationships and it aggregates more complex information from neighbours. MHAMFD considers compound semantic relationships from interweaving different levels of behavioural relationships, which improves the quality of the selected neighbours of the target node. The results showed that MHAMFD performed the best with the different training set regardless of the metric (i.e. F1, Recall and Precision).

**Table 3:** Anomaly detection effectiveness with balanced sample nodes

Train:Val:Test	1:1:3			2:1:2			3:1:1		
Metrics	F1	Recall	Precision	F1	Recall	Precision	F1	Recall	Precision
Metapath2vec	0.795	0.7191	0.8888	0.8037	0.7166	0.9148	0.7384	0.6857	0.8
GCN	0.6067	0.6175	0.6296	0.5737	0.5908	0.5894	0.6036	0.6304	0.6121
HAN	0.7658	0.7762	0.8051	0.7543	0.7695	0.796	0.7896	0.8025	0.8138
MHAMFD_hierar_	0.8014	0.8131	0.9022	0.8095	0.8658	0.9194	0.8662	0.8346	0.9143
MHAMFD_single_	0.7611	0.7917	0.8908	0.7979	0.8391	0.8948	0.7942	0.8417	0.9021
MHAMFD_multi_	0.7981	0.8014	0.8989	0.8048	0.8490	0.8748	0.7816	0.8392	0.8846
MHAMFD	0.8361	0.8764	0.9194	0.8679	0.8813	0.9386	0.8806	0.8692	0.9435

### Model analysis

**Fig. 5:**
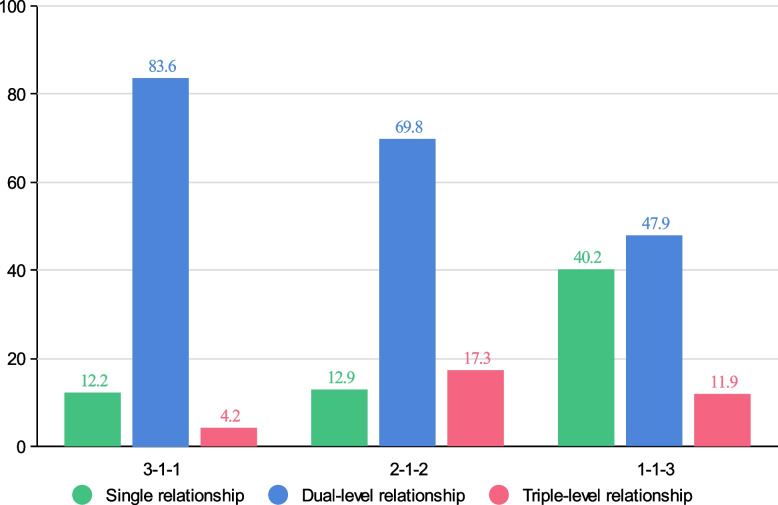
Attention values (%) for different levels of behavioural relationships

Some previous graph embedding models have been based on the assumption that the learning performance increases with the contribution of the path to node embedding. In a graph constructed for health insurance fraud detection, two patient nodes may be connected by more than a single-level behavioural relationship. For nodes that are intertwined and connected by multilevel behavioural relationships, the semantic relationships are often more complex. We argue that different levels of behavioural relationships have different degrees of influence on the final embedding of patient nodes. Figure 5 shows the results with the hierarchical attention mechanism, the dual-level behavior relationship has the greatest contribution, while the triple-level behavior relationship has the worst contribution. This shows that the neighbor nodes obtained from the dual-level behavior relationship are more similar to the target nodes and make greater contributions to the final task. The triple-level behavior relationship has the least influence, because the number of neighbor nodes connected by the target node through the triple-level relationship is far less than that of the single-level relationship and the dual-level relationship. The target node gets less information from neighboring nodes, which leads to the influence of the triple-level behavior relationship is not as good as that of the other two levels. The specific attention results are shown in Table 4. In the results of the attention value for behavior relationship, we can see that behavioral relationship PDTP has the greatest influence on node embedding in the dual-level behavior relationship. This means that the characteristics of patients who have seen a doctor in the same department on the same day are similar to each other and highly correlated. The table shows that the degree of attention may reflect the interpretability of the model. This can be used to select neighbour nodes according to the appropriate hierarchy of behavioural relationships.

**Table 4:** Attention result in Medical-1 datasets

Levels of behavioural relationship	Single relationship			Dual-level relationship			Triple-level relationship
Attention value for levels of behavioural relationship	0.129			0.698			0.173
Behavioural relationship	PDP	PTP	PMP	PDTP	PDMP	PTMP	PDTMP
Attention value for behavioural relationship	0.475	0.346	0.179	0.619	0.302	0.079	1

### Ablation experiment

**Fig. 6:**
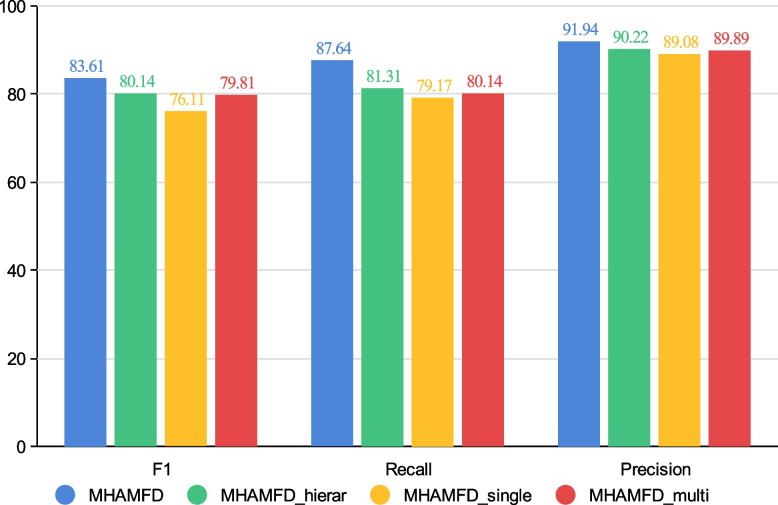
Experimental results (%) with different variants of MHAMFD

To validate each component of our proposed model, we performed further experiments with different variants of MHAMFD (see the ‘Baselines’ section). Figure 6 compares the performances of the different variants. The horizontal axis shows the variants and the vertical axis represents the performance. The performance was evaluated according to the following metrics: f1, recall and precision. The results are presented for 20%, 40% and 60% of the training set of Medical-2. Between MHAMFD$_{\text{ single } }$ and MHAMFD$_{\text{ multi } }$, the latter performed slightly better. This indicates that the multilevel behavioural relationship path contains more complex and accurate information about the nodes and confirms the accuracy of selecting neighbour nodes with different levels of behavioural relationships. The results for MHAMFD$_{\text{ hierar } }$, MHAMFD$_{\text{ single } }$ and MHAMFD$_{\text{ multi } }$ showed that the model performance was significantly improved by aggregating different levels of behavioural relationships simultaneously. Finally, the results for MHAMFD and MHAMFD$_{\text{ hierar } }$ showed that different levels of behavioural relationships had different impacts on the final task and that an attention mechanism was needed to learn these impacts and obtain better results.

### Visualisation

**Fig. 7:**
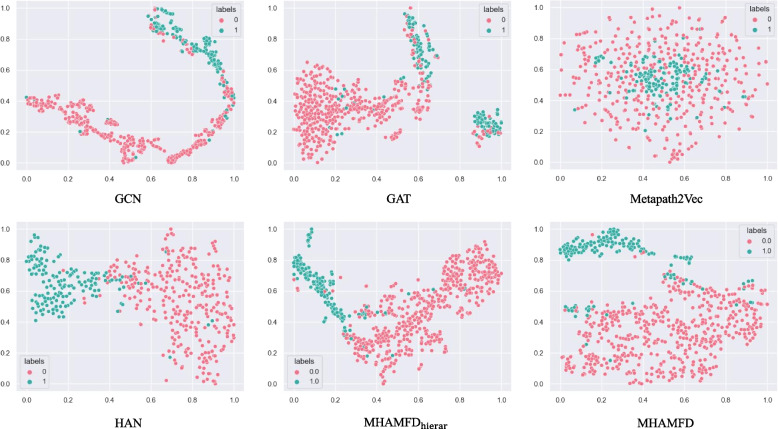
Visualisation of the learned node embeddings with the Medical-2 dataset

For a more intuitive comparison, we visualised the learned embeddings in two-dimensional space. This allows the node distribution to be represented in a low-dimensional space. We used t-distributed stochastic neighbour embedding (t-SNE) to visualise patient nodes embedded in Medical-2 for each model. For convenience of observation, we took all of the positive samples and randomly selected 500 nodes from the negative samples. Figure 7 shows the results. Metapath2vec performed poorly and could not distinguish the node distribution. GAT and GCN could mostly distinguish between positive and negative samples, but there was no sharp boundary and a considerable number of positive and negative samples were mixed together. HAN and MHAMFD$_{\text{ hierar } }$ perform better than GAT and GCN. This is because HAN used multiple meta-paths and thus could obtain more semantic information. Meanwhile, MHAMFD$_{\text{ hierar } }$ not only used single-level behavioural relationships to sample neighbour nodes but also added multilevel behavioural relationships to select appropriate neighbour nodes. Overall, MHAMFD performed the best. It clearly distinguished between different classes and there was a clear boundary between positive and negative samples. These results further illustrate the effectiveness of adding different levels of aggregation modules.

### Hyper-parameter analysis

**Fig. 8:**
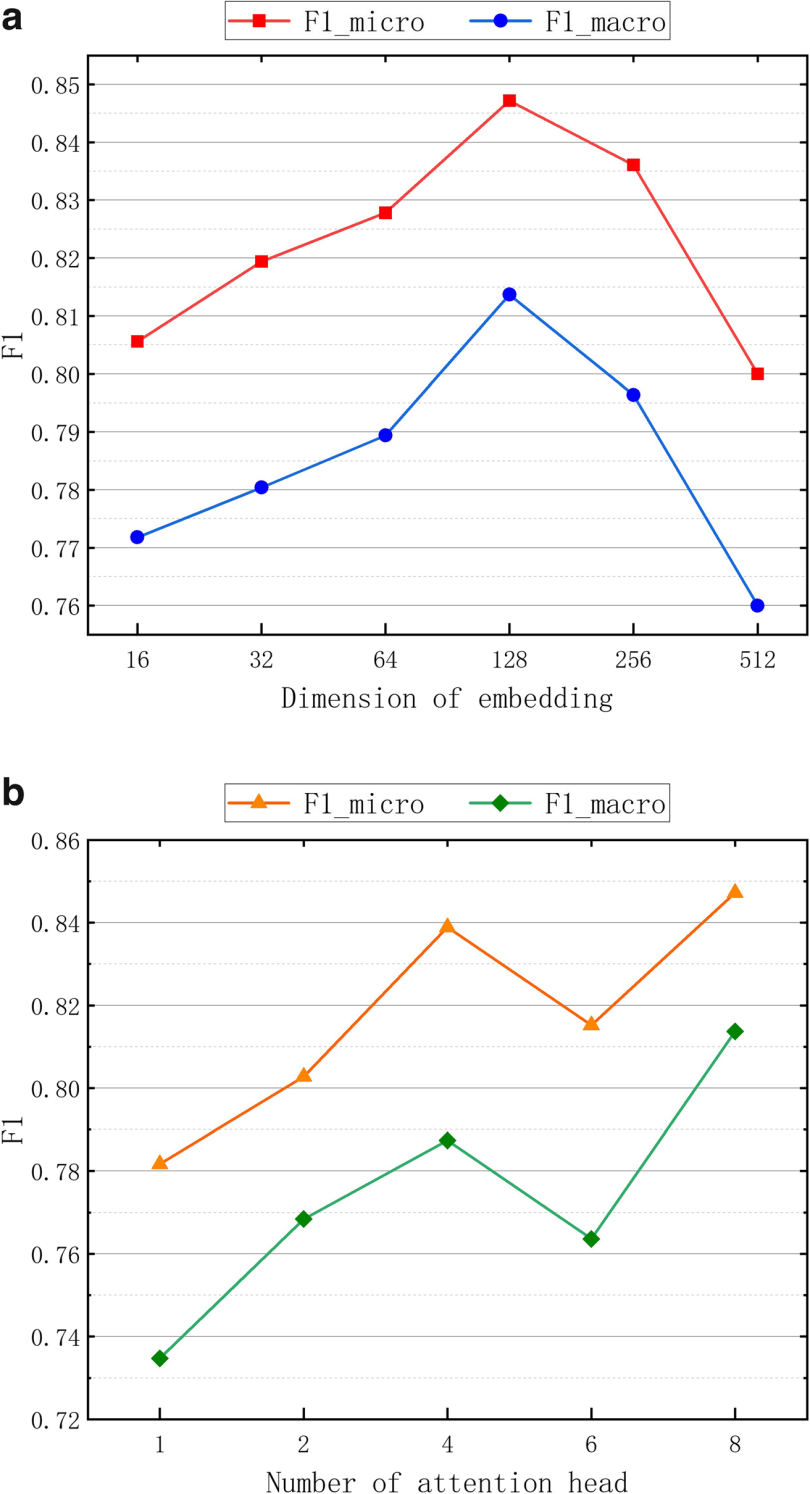
Hyper-parameter sensitivity analysis of MHAMFD: **a** Dimension of the final embedding *Z*; **b** Number of attention head *K*

We further studied the sensitivities of several key hyper-parameters by varying them in different scales. We first tested the effect of the dimension of the final embedding *Z*, and the result is shown in Fig. 8(a). We set the dimensional range of the final embedding *Z* to $Z \in (16,32,64,128,256,512)$. From Fig. 8(a), we observed that the performance of the model improves when the value of *Z* is gradually increased from 16 to 128. However, when the value of *Z* is increased further from 128, the performance of the model starts to decrease. This is because MHAMFD needs a suitable dimension to encode the behavioral relationship information. And as the dimension increases further, it may introduce additional redundancy. We explored and analyzed the performance of MHAMFD with various number of attention head. We set the value of *K* to $K \in (1,2,4,6,8)$ and the result is shown in Fig. 8(b). We observed that the performance of MHAMFD is basically improved as the attention head increases. Meanwhile, we also find that multihead attention can make the training process more stable.

## Conclusion

In this study, we considered the problem of health insurance fraud detection. We used a real healthcare dataset to explore different behavioural relationships of visits by patients. The impact of interactions between different objects in a healthcare scenario on fraud detection was analysed. These interactions were captured by an AHIN in a model called MHAMFD. Different levels of behavioural relationships are used to select appropriate neighbours, which considers the composite semantic information from the interweaving of different relationships and improves the quality of neighbour nodes. The embedding representation of target nodes is comprehensively learned by the aggregation of behavioural relationships within, between and at different levels. The effectiveness of MHAMFD at health insurance fraud detection was verified through experiments using real medical data. In future work, we will conduct explanatory studies on applying MHAMFD to health insurance fraud detection in different scenarios.

## Data Availability

The data that support the findings of this study are available on request from the corresponding author. The data are not publicly available due to privacy or ethical restrictions.

## Abbreviations

HIN: Heterogeneous information network
AHIN: Attributed heterogeneous information network
AGG: Aggregation
